# Supplementary material for: Comprehensive Omics Analysis Reveals Cold-Induced Metabolic Reprogramming and Alternative Splicing in Dendrobium officinale
Source: Plants (Basel). 2025 Jan 30;14(3):412. doi: 10.3390/plants14030412 (PMC11820321; doi:10.3390/plants14030412)
Supplement: Supplementary file 1 [file plants-14-00412-s001.zip › plants-3438627-supplementary/Supplementary_Figure.pptx]

## Slide 1
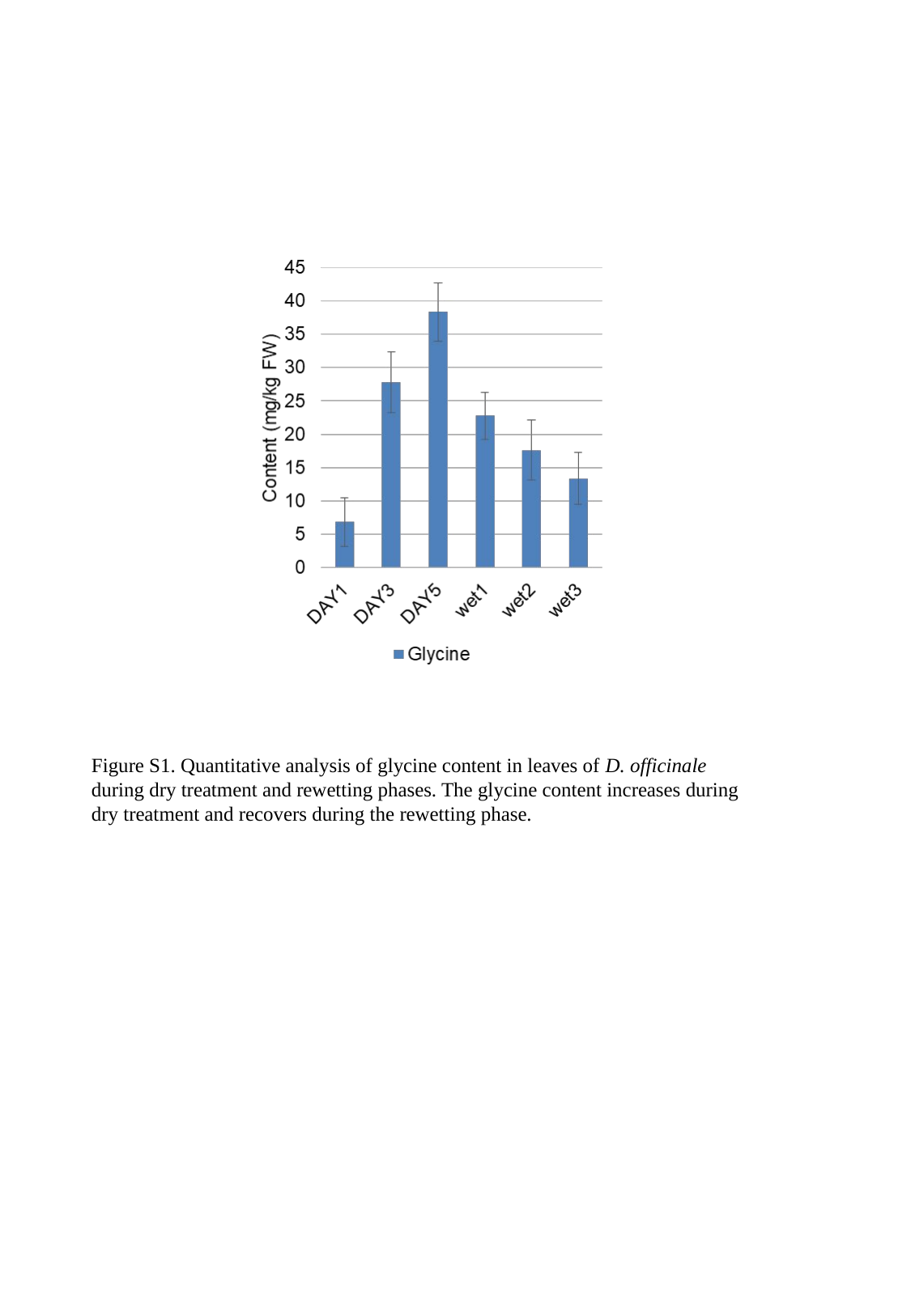

Figure S1. Quantitative analysis of glycine content in leaves of D. officinale during dry treatment and rewetting phases. The glycine content increases during dry treatment and recovers during the rewetting phase.

## Slide 2
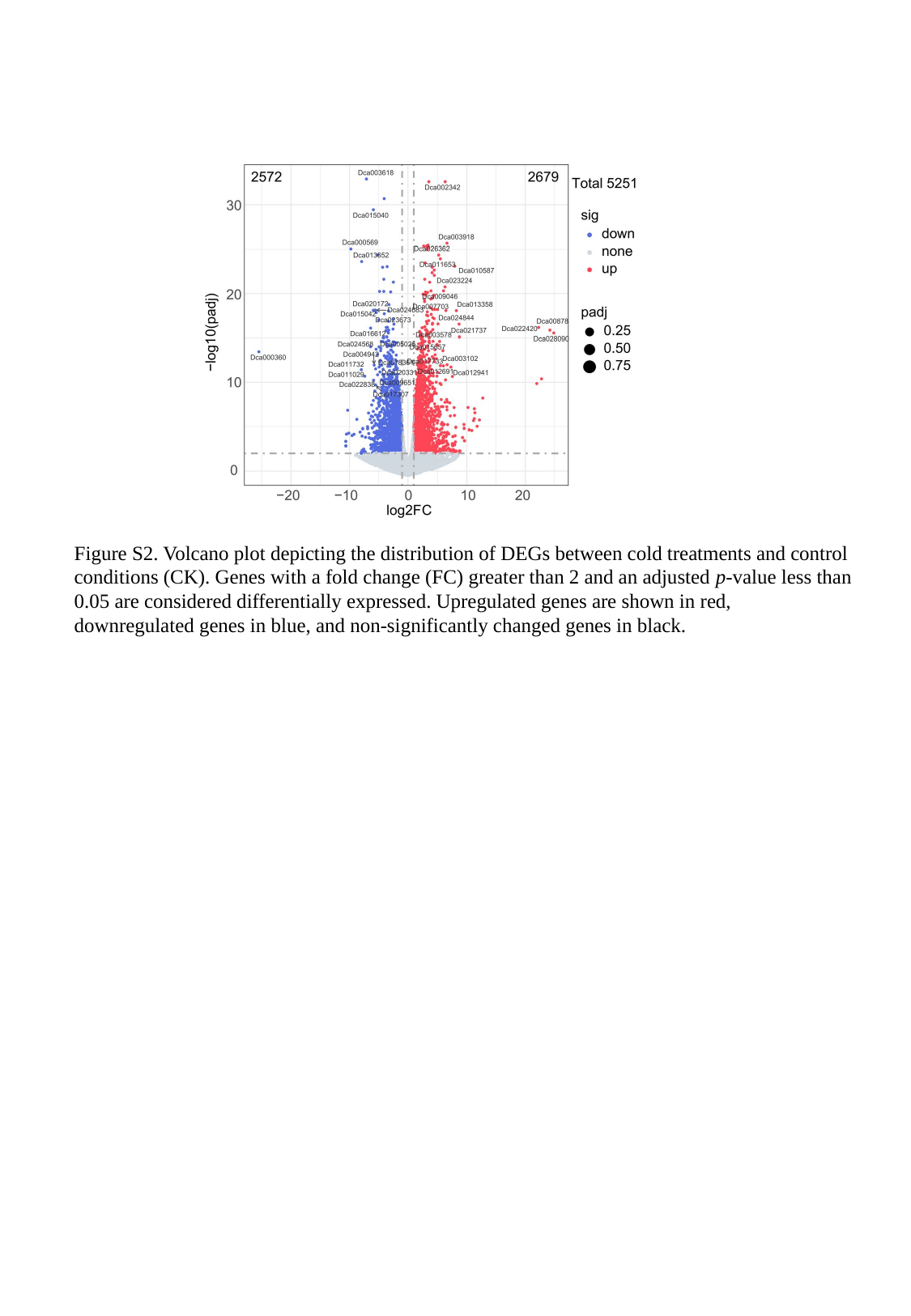

Figure S2. Volcano plot depicting the distribution of DEGs between cold treatments and control conditions (CK). Genes with a fold change (FC) greater than 2 and an adjusted p-value less than 0.05 are considered differentially expressed. Upregulated genes are shown in red, downregulated genes in blue, and non-significantly changed genes in black.

## Slide 3
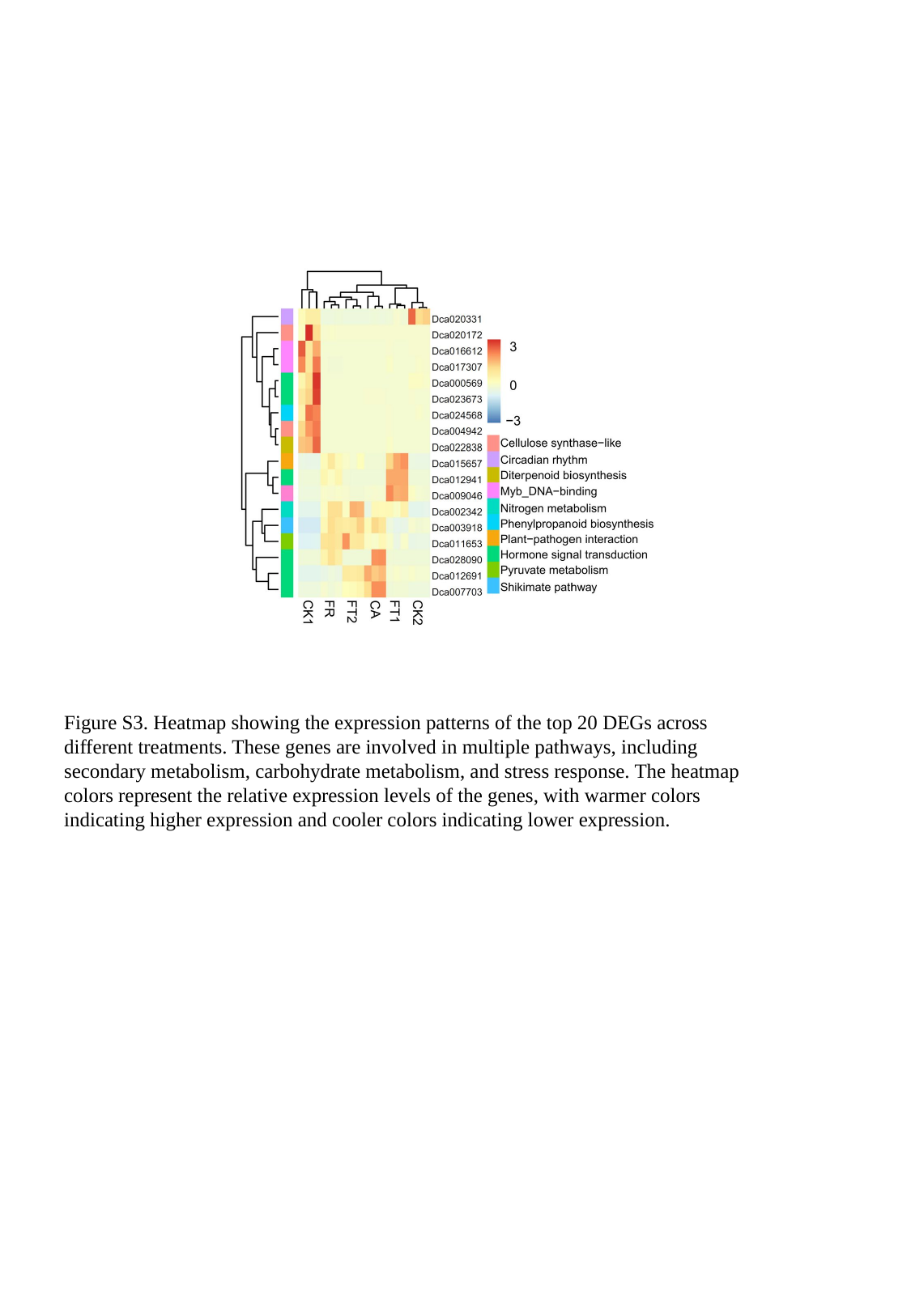

Figure S3. Heatmap showing the expression patterns of the top 20 DEGs across different treatments. These genes are involved in multiple pathways, including secondary metabolism, carbohydrate metabolism, and stress response. The heatmap colors represent the relative expression levels of the genes, with warmer colors indicating higher expression and cooler colors indicating lower expression.

## Slide 4
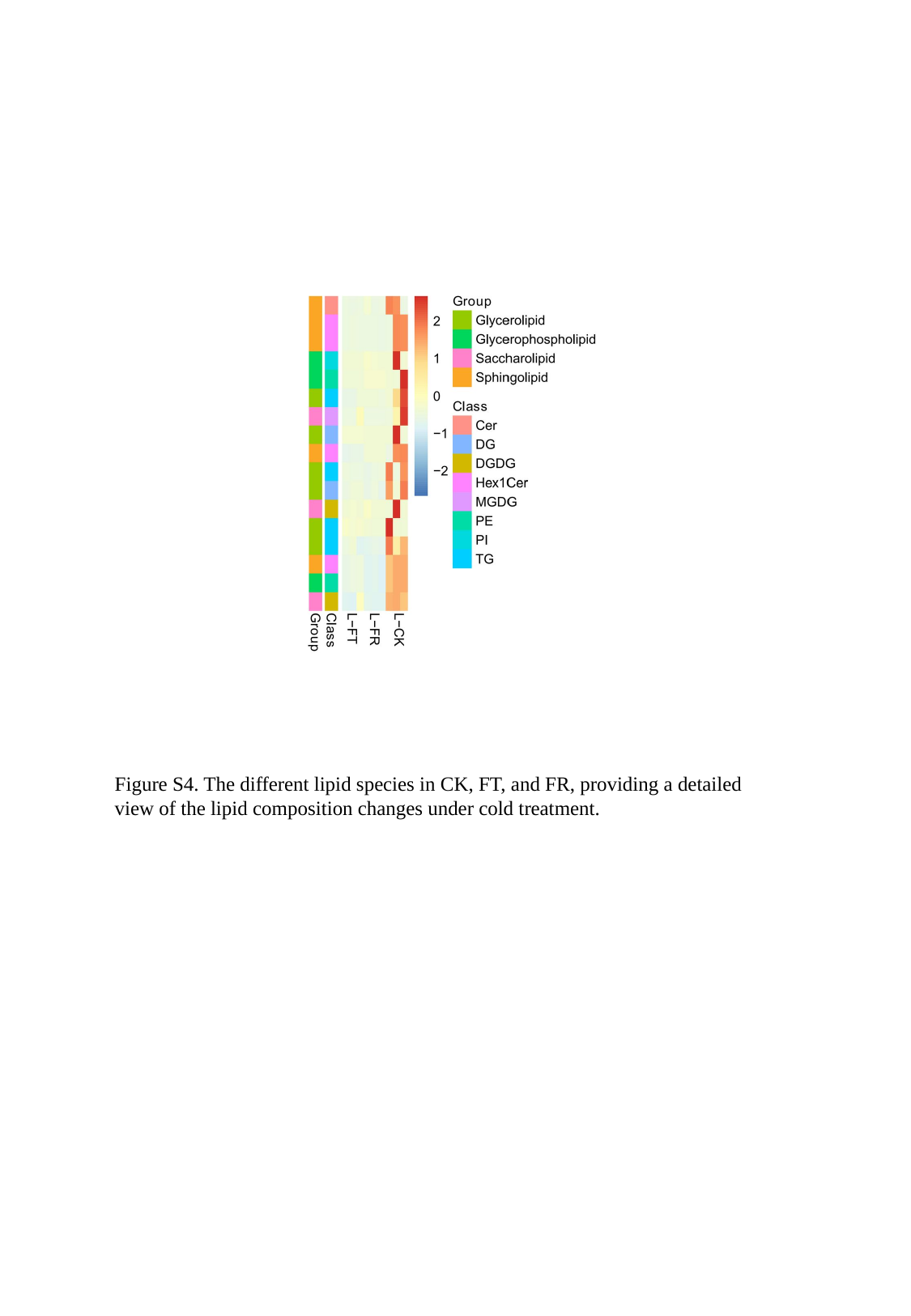

Figure S4. The different lipid species in CK, FT, and FR, providing a detailed view of the lipid composition changes under cold treatment.

## Slide 5
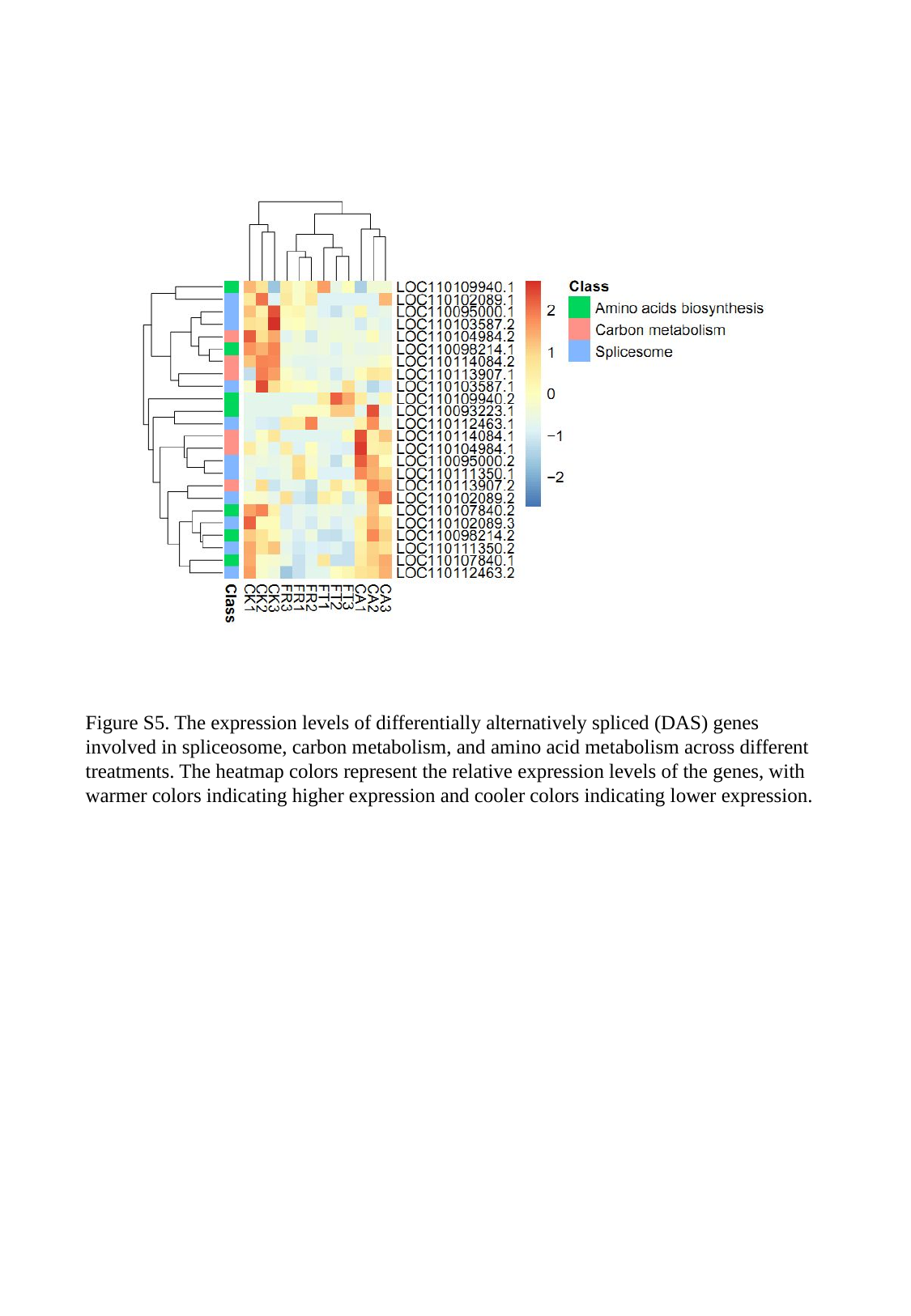

Figure S5. The expression levels of differentially alternatively spliced (DAS) genes involved in spliceosome, carbon metabolism, and amino acid metabolism across different treatments. The heatmap colors represent the relative expression levels of the genes, with warmer colors indicating higher expression and cooler colors indicating lower expression.

## Slide 6
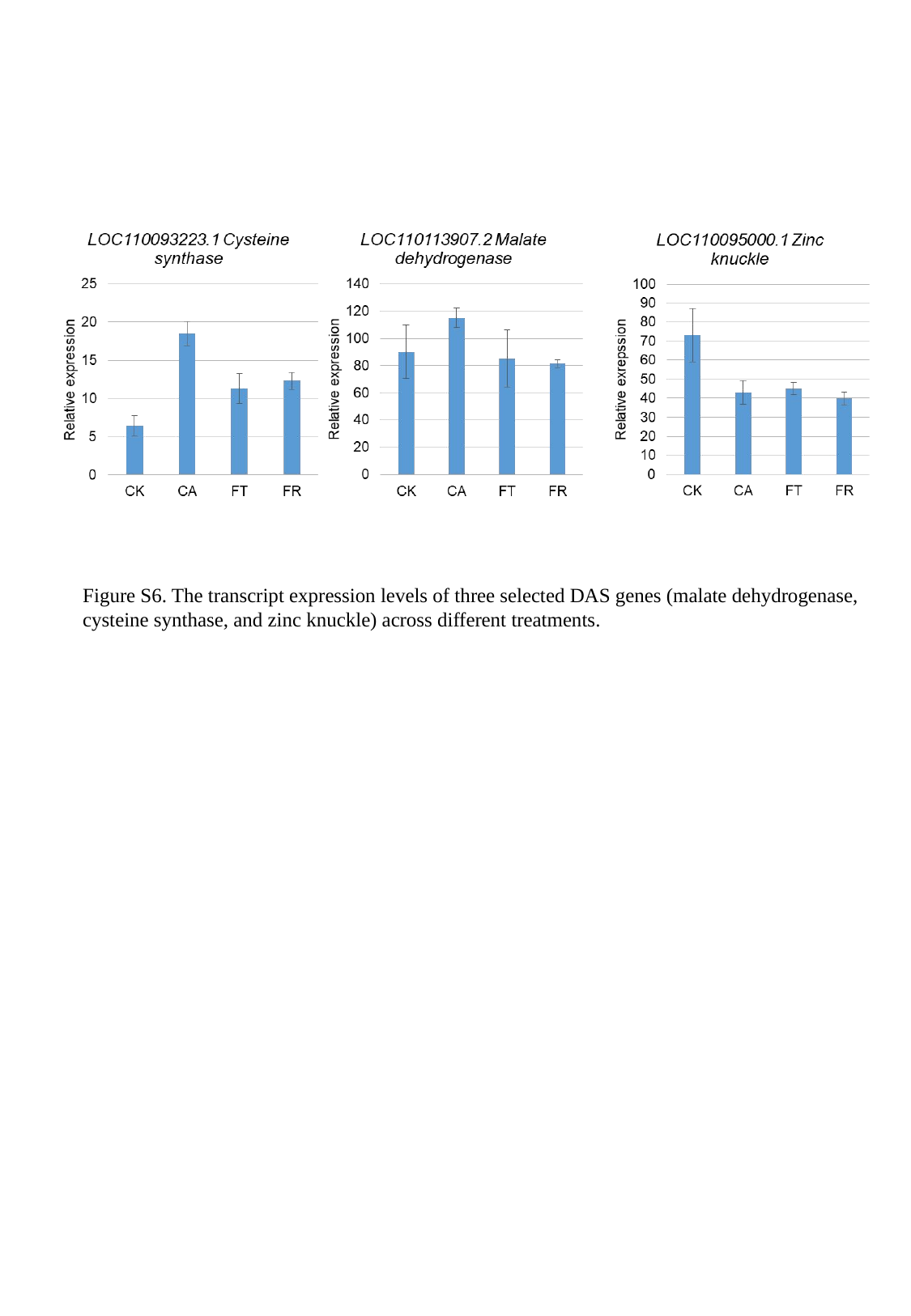

Figure S6. The transcript expression levels of three selected DAS genes (malate dehydrogenase, cysteine synthase, and zinc knuckle) across different treatments.

## Slide 7
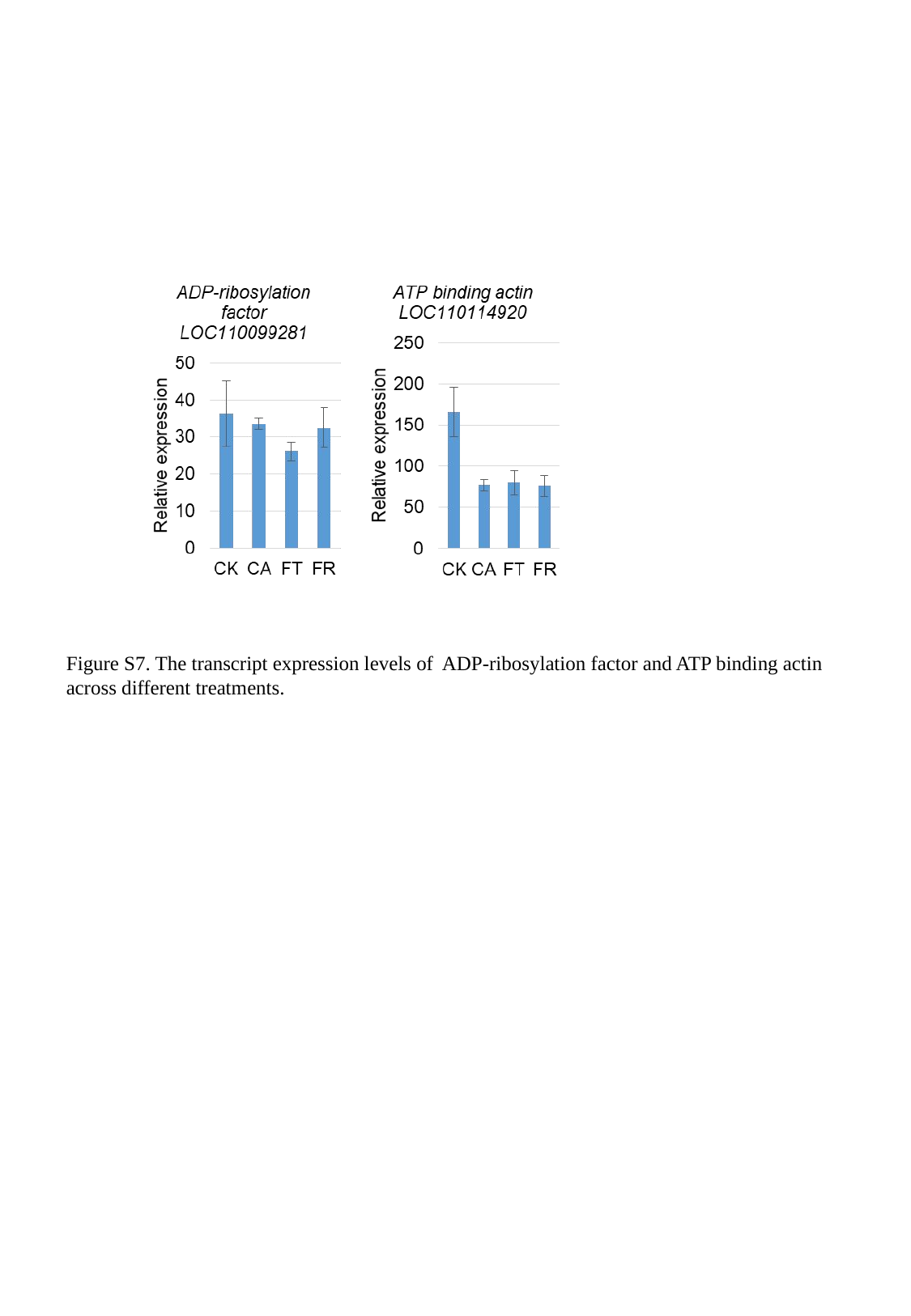

Figure S7. The transcript expression levels of ADP-ribosylation factor and ATP binding actin across different treatments.

## Slide 8
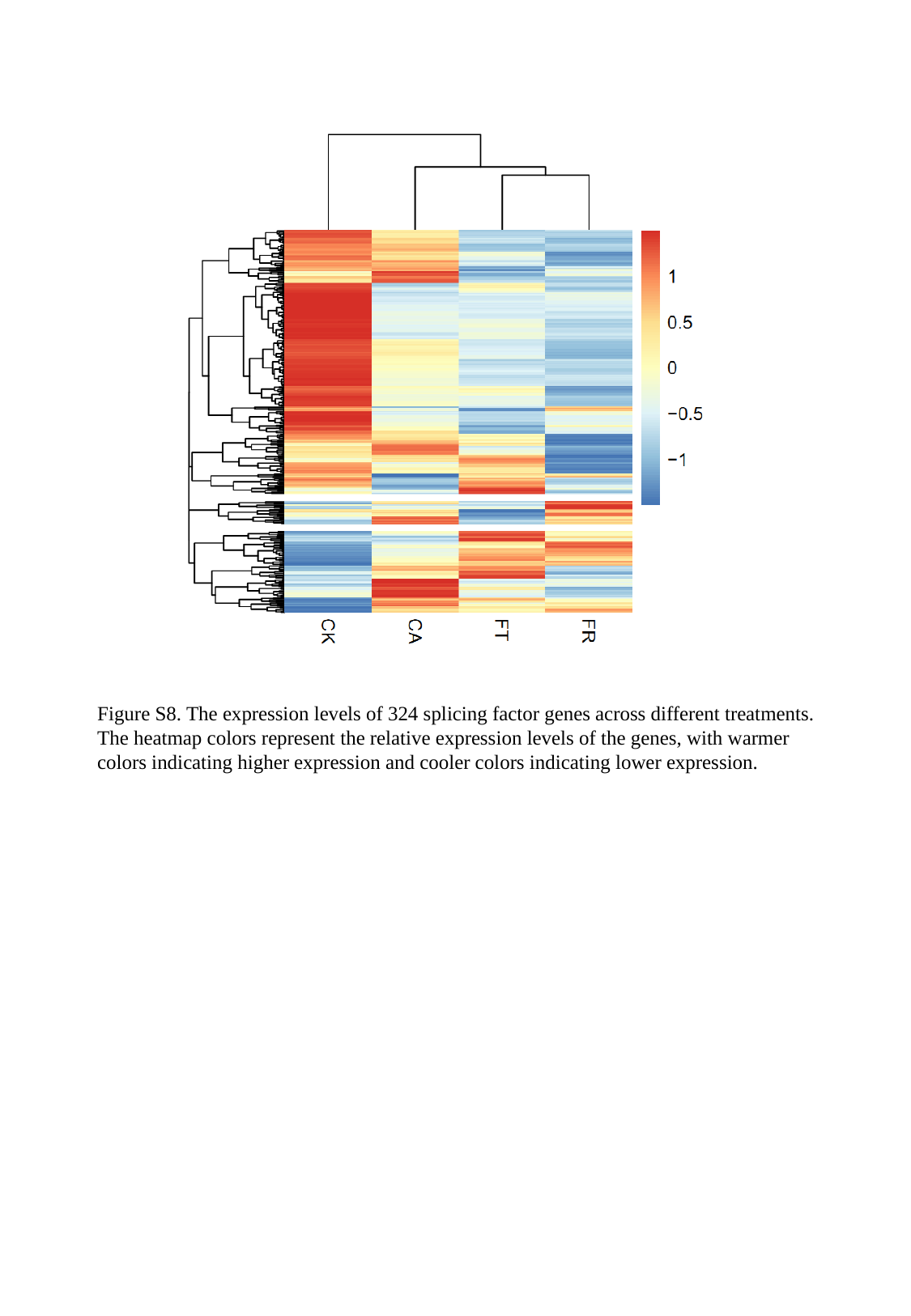

Figure S8. The expression levels of 324 splicing factor genes across different treatments. The heatmap colors represent the relative expression levels of the genes, with warmer colors indicating higher expression and cooler colors indicating lower expression.

## Slide 9
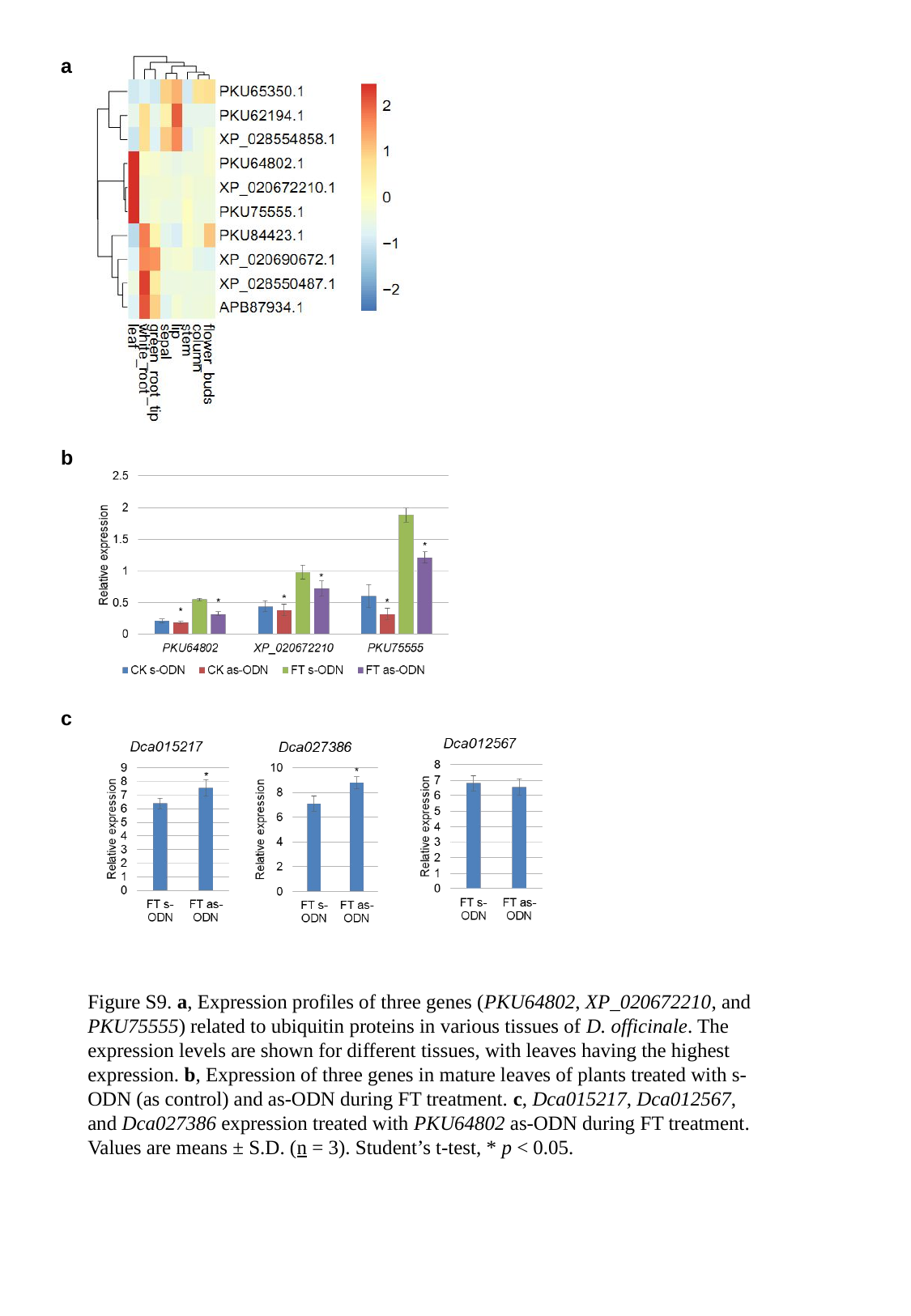

a
b
c
Figure S9. a, Expression profiles of three genes (PKU64802, XP_020672210, and PKU75555) related to ubiquitin proteins in various tissues of D. officinale. The expression levels are shown for different tissues, with leaves having the highest expression. b, Expression of three genes in mature leaves of plants treated with s-ODN (as control) and as-ODN during FT treatment. c, Dca015217, Dca012567, and Dca027386 expression treated with PKU64802 as-ODN during FT treatment. Values are means ± S.D. (n = 3). Student’s t‐test, * p < 0.05.
